# Children's Understanding of Their Illness

**Published:** 1988

**Authors:** Carol Kendrick

**Affiliations:** Bristol Children's Hospital


### Childhood Cancer: The Patients' Understanding of Disease and Treatment

**Carol Kendrick**

Bristol Children's Hospital

Over the past two decades, general acceptance has been gained for the idea that children with cancer should be told about their disease (Agranoft and Mauer, 1965). There is still, however, a relative dearth of systematic knowledge about how young children comprehend or make sense of this well intentioned open approach. (Brewster 1982, Eisler 1984).

In this preliminary study setting out to describe young children's understanding of their disease, three principal questions are addressed:

1) How do children in hospital acquire knowledge about cancer?
2) What do young children understand about their own illness and its treatment?
3) To what extent is comprehension important?

### METHOD

a) *Sample*
There were 25 children (13 boys and 12 girls) in the study, with an age range of 2–11 years. Ten of these children had leukaemias, eleven had solid tumours, and four, brain tumours. Seven of the children were newly diagnosed and were observed over the first month post-diagnosis.

b) *Procedures*
The techniques used to obtain information about the children's understanding of their illness were:

1) direct unstructured observations of the children in hospital by a participant but non-directive observer;
2) informal interviews with the children in which they were encouraged to talk about what was wrong with them, and about the treatment they were receiving. It had already been established that the interviewer/observer (CK) had come to the unit in order to learn about chilhood cancer, and thus the children, as veterans, were in a position to provide her with information.

c) *Time Sampling*
The material in this study was all gathered from the Bristol Royal Hospital for Sick Children between July and September 1984. The observations and interviews spanned across playtimes, treatments (bone marrow tests, lumbar punctures, X-rays and radiotherapy), meal times, doctors' rounds, friends' visiting times and television watching, in an attempt to get an overall picture of the children's world in the hospital. The median time spent with each child was 4 hours (range 1–15 hours).

### HOW THE CHILDREN LEARNT ABOUT CANCER

All the children in the study were initially told that they had cancer by one of the consultants on the unit (M.M or T.O.) or by their own parents, who had previously talked with a consultant. During this initial explanation the following areas were dealt with: which part of the body has something wrong with it; how this was discovered; what is going to be done about this; and how will it affect you (the child). In all cases the actual name of the illness, i.e. leukaemia, lymphoma, was used. This relatively formal occasion was rapidly followed by a series of informal encounters with other people in the hospital. Without exception, the newly diagnosed children were seen to be actively seeking out information at this stage, and indeed, appeared to be extra-receptive to any cues regarding their illness. Within one week of hearing their diagnosis the children all showed a marked increase in their vocabulary, i.e. using newly acquired words such as 'platelets', 'radiotherapy' and 'biopsy' in an appropriate manner, as well as acquiring a considerable knowledge of physiology and pathology.

FOR EXAMPLE:
Marlene (8 years) when first told that she had leukaemia did not know anything about the functions of blood in her body. She did know the colour of blood, but nothing else. By the end of the first week she had grasped the basic concept that different 'bits' of blood perform different functions and that in her own case these functions were not being carried out adequately.

The children were therefore picking up information which was of immediate relevance to themselves. They were learning about cancer from all those around at the time: the physiotherapists, nurses, play-leaders, domestic staff, and most importantly, the other patients on the ward (other children with cancer) and their own parents. It should be stressed that this information came in the form of non-verbal as well as verbal messages. Games, pictures, puppets and observations of the hospital routine were clearly contributing to the children's fact finding ventures. The very first experience of any treatment or procedure was quite understandably of great importance in establishing a pattern for future occasions.

FOR EXAMPLE:
Imra (6 years) was fortunate enough to go for her first radiotherapy session alongside Kirsty (4 years). Kirsty, who had already experienced several treatments, took the situation in hand. She introduced Imra in a matter-of-fact way to the staff, the equipment and toys at the radiotherapy centre and then calmly proceeded to settle down for her own treatment. Imra henceforth looked forward to her visits to the RT centre.

### THE CHILDREN'S UNDERSTANDING OF THE SITUATION

a) *Who does what in a hospital*
This potentially complex area of institutional life was grasped rapidly by even the youngest of the children in the sample. None of the children had previously been in-patients but they were able to differentiate with perfect ease between nurses, doctors, domestic workers, and to predict which jobs would be done by whom, and to reconstruct the hospital routine.

FOR EXAMPLE:
Paul (9 years) called imperiously, "Nurse, nurse, I want a bed pan," whilst surrounded by doctors on a ward round. He had ascertained quite correctly that this was a duty not usually carried out by doctors. Kirsty (4 years), going in search of some plasticine, directly approached the playleader rather than asking the junior doctor sitting next to her.

*b) The necessity for treatment*

The question of why they were having to undergo restricting, uncomfortable, and in some cases painful procedures in order to treat their illness was accepted and understood, at least at some level, by the children. In some cases there was a sort of fateful unquestioning acceptance.

FOR EXAMPLE:

Lynette (5 years), after completing a course of chemotherapy, asked quite nonchalantly when her sister (who was perfectly healthy) would be coming for her treatment.

The parents' urgency and insistence seemed to communicate to the children the importance of the treatment, bringing with it a change in behaviour.

FOR EXAMPLE:
Thornton (2 years), who had at first refused point blank to take his Prednisolone tablets, much to his parents' distress, was within a few days submitting to this indignity.

Ruth, also aged 2 years, took her tablets as required in quite a docile manner which contrasted strongly with her fussing, clinging and generally difficult behaviour if called upon to do anything non-essential to life, such as washing or getting dressed.

*c) Key words and crucial indicators*

After diagnosis the children quickly came to learn the importance of various key words and crucial indicators in their new world.

Certainly the children aged five and upwards could appropriately categorise as good or bad words such as neutropenic, remission, relapse and pyrexia. Along with their parents, these children could also be seen anxiously awaiting the results of vital tests such as bone marrows and biopsies. These somewhat abstract indicators of improvement and decline in health carried even more weight than how the child was actually feeling.

FOR EXAMPLE:

| | |
|---|---|
| Nina (9 years) | "I'm all hot and sticky but I haven't got a temperature." |
| Annie (8 years) | "The X-ray said the lump was still there even though you can't feel it any more." |

*d) Cause and effect*

The link between chemotherapy and changes in appearance such as hair loss and puffy cheeks were accurately understood and verbalised by the children of five years and over.

FOR EXAMPLE:

Natasha (9 years), when asked directly by two visitors to the ward why her hair was so thin, replied "It's all come out because of my treatment, the Vincristine does that."

Lynette (5 years) was being sick whilst the observer held a bowl for her, making sympathetic noises. Lynette remarked "I'm on my treatment (chemotherapy) again, that's why."

The older children, in trying to explain their disease during interviews, often sounded like research scientists.

FOR EXAMPLE:
Paul (9 years): "My cancer is a disease where some of the blood cells that fight disease don't grow properly. This means that resistance to any kind of illness in my body is extremely low."

### DEVELOPMENT AND REGRESSION

Many of the children appeared to be exhibiting an understanding of their own disease which far exceeded expectations for their age and stage of development. Most noticeably the youngest children in the sample showed a marked acceleration in their cognitive development. The use of new words and ideas has already been mentioned. In addition, a mature understanding and empathy for other children going through the same experiences as themselves was shown.

FOR EXAMPLE:
Jenny (6 years), upset at the prospect of having a central venous catheter inserted, was comforted by Lorraine (5 years). Lorraine, who already had a line in situ, said "It's just like a worm . . . and you don't feel it going in."
However, at the same time as these advances, the children were also showing all the classic signs of regression seen in hospitalised children, such as wanting to be dressed and fed, returning to a bottle or dummy, and wetting the bed at night.

### IS IT IMPORTANT FOR CHILDREN TO UNDERSTAND?

Observations of children in this study point to some of the possible reasons why it is vital for children with cancer to understand about their illness.
a) Misunderstandings were associated with high levels of anxiety for the children.

FOR EXAMPLE:
Malcolm (9 years), having been told that he was going to be 'locked off' from his drip for the afternoon, looked distinctly uneasy.
b) By coming to understand why they were receiving treatment, children realised that they were special, and cared for, both by their parents and hospital staff. They were not simply being punished for being ill in the first place. An understanding of the situation allowed a determined compliance on the part of the child to necessary yet unpleasant procedures.

FOR EXAMPLE:
Thornton (2 years) unhesitatingly held out his hand for a blood test whilst at the same time crying at the prospect of a painful needle prick.
c) The fact that children with cancer are contributing to each other's understanding makes it doubly important that each child should understand. Misunderstanding could potentially spread amongst the children on a ward.

### CONCLUSION

From this preliminary descriptive study looking at the ways in which children with cancer obtain an understanding of their disease, several practical messages emerge of relevance to anyone in contact with these children.
1) The very early days after diagnosis and the initial experiences of hospital procedures are vital. It is especially at this stage, when the child is actively seeking out clues about hospital life, illness and treatment, that coherent and non-contradictory messages are vital. Good communication between all hospital staff in contact with children and their families can go some way towards ensuring this.

2) The evidence that children's ability to understand is linked with their age and stage of development (Bibace & Walsh, 1980) is reinforced by this study. Whereas the under-fives perceive their world in very concrete terms, the older children grasp abstract concepts of categorisation. Children with cancer do make dramatic leaps forward in terms of their intellectual functioning which coincides with the idea of accelerated developmental spurts seen at times of stress by Anna Freud (Freud, 1965). However, alongside the children's slick use of medical jargon there may be concealed areas of ignorance. Regression in other aspects of children's behaviour should also be anticipated and dealt with calmly.

3) Young children's understanding often comes from direct first hand experience and their play situations. With sensitive staff available, these opportunities can be utilised as moments for learning. For example, a playroom equipped with 'toy' IV drips, radiotherapy machines, nurses' uniforms and so on can facilitate the exploration of such areas.

4) It is important for children with cancer to understand. Misunderstandings can lead to unhappiness and anxiety which quickly transmits itself to other children in the ward. An understanding of the necessity for treatment enables better compliance and appreciation of the care that is being lavished on the child even during times of pain and discomfort.

### REFERENCES

AGRANOFF, J. H. and MAUER, A. M. (1965) What should the child with Leukemia be told? *American Journal of Diseases of Children* **110**, 23.

BIBACE, R. and WALSH, M. E. (1980) Development of children's concepts of illness. *Paediatrics* **66**, 912–917.

BREWSTER, A. B. (1982) Chronically ill children's concepts of their illness. *Paediatrics* **69**, 355–362.

EISLER, C. (1984). Communicating with sick children, *Journal of Child Psychology & Psychiatry* **25**, 181–189.

FREUD, A Normality and Pathology in childhood. New York: International Universities Press. 1965.

### ACKNOWLEDGEMENTS

This study was funded by the Dorothy Fielding Trust. It was made possible by the co-operation of the children and their parents and the support of members of the paediatric oncology unit.

For further details of the study see also:
Kendrick, C., Culling, J., Oakhill, T. & Mott, M. Children's understanding of their illness and its treatment within a paediatric oncology unit. ACPP Newsletter, 1986, Vol. 8, No. 2, 16–20.

